# Barriers and enablers shaping autistic college students’ pathways into advanced manufacturing careers

**DOI:** 10.1080/00049530.2026.2746627

**Published:** 2026-10-15

**Authors:** Nigel Newbutt, Yujiao Fan, Hyo Kang, Ann-Marie Orlando, Navid Asadizanjani, Adam Nazef

**Affiliations:** a School of Teaching and Learning, University of Florida, Gainesville, FL, USA; b Digital Worlds, University of Florida, Gainesville, FL, USA; c Center for Autism and Related Disabilities, University of Florida, Gainesville, FL, USA; d Florida Semi-Conductor Institute, University of Florida, Gainesville, FL, USA

**Keywords:** Workplace, employment, autistic college students, advanced manufacturing

## Abstract

**Objective:**

Autistic college students are strongly represented in STEM disciplines, yet continue to experience disproportionately poor employment outcomes after graduation. This study explores their lived experiences navigating educational and career pathways into advanced manufacturing, a high-demand sector facing persistent workforce shortages and substantial public investment, yet largely absent from autism and employment scholarship to date.

**Method:**

Sixteen autistic STEM undergraduates (current or previous) from across the US participated in three 90-minute focus groups conducted via Zoom. Sessions examined students’ career aspirations, job search experiences, perceived barriers, and support needs, and were analysed using thematic analysis to identify enablers and systemic barriers shaping participants’ transitions from education to industry.

**Results:**

Participants demonstrated proactive engagement in developing technical skills through coursework, self-directed learning, and hands-on experiences. However, they encountered opaque hiring processes, unclear interview expectations, limited access to prior industry experience, workplace sensory challenges, and fragmented institutional support, often relying on informal networks and AI-based tools to compensate. Participants emphasized inclusive, sensory-aware learning environments, structured career preparation, and autism-informed mentorship as critical bridges between academic training and workforce expectations.

**Conclusions:**

Employment disparities reflect systemic misalignment rather than individual deficits, underscoring the need to redesign education-to-work pathways that recognize and leverage autistic strengths.

## KEY POINTS

**What is already known about this topic:**

1. Autistic college students are strongly represented in STEM education.
2. Autistic graduates often face poorer employment outcomes than their peers.
3. Career and transition support for autistic students are often fragmented and poorly aligned.

**What this topic adds:**

1. Autistic college students build career readiness through coursework, self-learning, and hands-on experiences.
2. Opaque hiring processes and sensory demands create major barriers for autistic students to advanced manufacturing careers.
3. Neuroinclusive mentoring, clearer career preparation, and structured supports may help to improve pathways into work.

## Introduction

One in 31 children in the United States are diagnosed with autismOne in 31 children in the United States is diagnosed with autism (Shaw et al., 2025), and the number of autistic students entering higher education continues to grow (Barnhill, 2016; Davis et al., 2021). Drawing on multiple national and institutional datasets, Cox et al. (2026) estimate that 42.9%–47.0% of autistic high school students transition to college, that between 0.3% and 4.7% of all U.S. undergraduates are autistic (approximately 135,400–286,254 students), and that 2.8%–4.0% of students registered with Disability Services Offices are autistic. Even so, earlier estimates suggest that only 30% of autistic students had attended a 2- or 4-year college (Roux et al., 2015), leaving them underrepresented relative to peers with other disabilities. Among those who do enrol, autistic college students are disproportionately represented in STEM fields, with 34% pursuing STEM majors (Wei et al., 2013). Roughly 2 million autistic adults in the United States now hold post-secondary degrees, and autistic undergraduates enter STEM at rates above the national average of 22.8% (C. Anderson et al., 2021). Together, these patterns position autistic college students as a substantial, STEM-concentrated population whose educational and career pathways warrant focused attention whose educational and career pathways warrant focused attention.

Autistic students also bring important strengths to higher education and STEM. Research highlights strong technological aptitude, advanced writing skills, and intellectual self-confidence (A. H. Anderson et al., 2020; Sturm & Kasari, 2019), alongside qualities such as pattern recognition, data precision, and sustained focus (Doyle et al., 2022; Kazhine & Hadem, 2025). Several of these strengths align particularly well with the task demands of the advanced manufacturing sector, a career domain that integrates advanced technologies across production systems, equipment maintenance, and quality control processes (National Center for ONET Development, 2026; Unnithan & Janardhanan, 2026). For example, pattern recognition and data precision are critical skill sets for quality assurance and yield analysis; sustained focus and tolerance for repetitive, protocol-driven work map onto process engineering and fabrication; and systematic reasoning maps onto root-cause analysis and equipment diagnostics (Damanpak Rizi et al., 2023). Notably, these are areas in which the industry continues to face persistent labour and skills shortages, a challenge explicitly recognized in recent U.S. workforce initiatives under the CHIPS and Science Act (Damanpak Rizi et al., 2023; U.S. Congress, 2022). Where broader STEM contexts sometimes conflate technical strengths with expectations of extensive social performance, advanced manufacturing frequently rewards technical depth and procedural fidelity directly, making it a domain in which autistic strengths are not incidental to the work but constitutive of it (Cope & Remington, 2022; Tomczak, 2022). At the same time, these strengths are often constrained by institutional and relational barriers across higher education and employment. Autistic college students report challenges related to sensory sensitivities, communication, social interaction, time management, self-advocacy, and mental health (A. H. Anderson et al., 2017, 2018; Davis et al., 2021; Sturm & Kasari, 2019). Many of these difficulties are produced or intensified by environments not designed with autistic learners in mind (Cox et al., 2021). STEM contexts can be especially difficult when organizational cultures remain non-inclusive of autism, limiting institutions’ ability to recognize autistic talent and foster educational innovation (Matthews et al., 2024).

These problems extend beyond the classroom and into students’ early encounters with employment. Trusson and Travers (2025) show that university employability support is often experienced as bureaucratic, compartmentalized, and poorly coordinated, with weak alignment between disability services and careers or placement teams and declining support during off-campus internships (Perkins, 2024). Autistic students themselves can identify both the shortcomings of these systems and practical ways to improve them (Janse van Rensburg & Liang, 2025).

The higher-education barriers autistic students experience are mirrored in the labour market. Despite strong technical foundations, 59% of autistic college graduates struggle to secure their first full-time paid job (C. Anderson et al., 2021). This gap is increasingly understood not as a reflection of limited ability, but of long-standing structural barriers including hiring systems that disadvantage neurodivergent applicants (Davies et al., 2023; Wiggleton-Little & Callender, 2023), workplaces poorly matched to sensory and social processing differences, limited visibility of inclusive career pathways, and workplace cultures that equate cognitive difference with unreliability (Ezerins et al., 2024; Stockwell et al., 2025). These general patterns take on specific forms in advanced manufacturing. Cleanroom and fabrication environments impose sensory conditions, protective equipment, continuous machine noise, controlled and often glare-heavy lighting, and shift work, that can either suit or acutely disadvantage autistic workers, yet are rarely discussed with candidates in advance. Entry pipelines that depend on apprenticeships, security clearances, and industry certifications add procedural and disclosure hurdles beyond the standard interview, and shift-based team structures place particular weight on unwritten social protocols during handovers. Each of these is a sector-specific manifestation of the broader structural barriers documented above, not a generic employment barrier.

The double empathy problem (Milton, 2012) remains insufficiently addressed at the organizational level (Dimitriou et al., 2026), with the burden of repairing cross-neurotype communication often placed on autistic employees rather than on organizations, supervisors, and systems. Performance appraisal systems are also typically calibrated to neurotypical norms, while the long-term effects of feedback, compensation structures, and leadership pathways for autistic employees remain underexplored (Ezerins et al., 2024). As a result, unemployment and underemployment among autistic graduates are better understood as signs that higher-education pipelines, labour markets, and hiring cultures continue to operate on an exclusionary template (Hora, 2020; Sellergren, 2024). This calls for a shift away from deficit narratives and towards structural redesign that recognizes and leverages autistic strengths while reducing the procedural and social barriers that obscure them (Singh, 2024).

These issues are particularly urgent in the advanced manufacturing sector, which is facing major talent shortages, particularly as the microelectronics and semiconductor industry plays a central role in U.S. economic competitiveness and national security (Asadizanjani et al., 2021), yet the industry continues to face shortages of domestic talent, especially in quality assurance and process engineering roles (Damanpak Rizi et al., 2023). These issues are particularly urgent in advanced manufacturing: the microelectronics and semiconductor industry plays a central role in U.S. economic competitiveness and national security (Asadizanjani et al., 2021), yet continues to face shortages of domestic talent, especially in quality assurance and process engineering roles (Damanpak Rizi et al., 2023). Employers continue to struggle to recruit qualified entry-level workers in part because undergraduates often have limited exposure to microelectronics careers and few opportunities to build industry-relevant skills, despite major federal investment through the CHIPS Act (U.S. Congress, 2022; White House, 2022). Comparable policy initiatives elsewhere reinforce the global significance of this workforce challenge: the European Chips Act mobilizes more than €43 billion to expand semiconductor production and skills capacity (European Commission, 2023), while the United Kingdom’s National Semiconductor Strategy commits up to £1 billion over ten years towards semiconductor design, compound semiconductors, and workforce development (UK Government, Department for Science, Innovation and Technology, 2023). Together, these developments create both an urgent labour-market need and a strategic opportunity to build more inclusive pathways into high-impact technical sectors. Advanced manufacturing is not simply a subset of STEM employment; rather, it is a distinct occupational context whose features shape the barriers and enablers autistic students may encounter in specific and identifiable ways. Unlike much of software or research-based STEM work, advanced manufacturing, including semiconductor fabrication, microelectronics, precision engineering, and additive manufacturing, is characterized by (a) highly structured, protocol-driven task environments where precision, repeatability, and adherence to specification are core competencies rather than peripheral virtues; (b) distinctive sensory environments including cleanrooms, fabrication floors, and shift-based operations with continuous machine noise, controlled lighting, protective equipment, and restricted movement; (c) credentialing and hiring pipelines that rely heavily on internships, apprenticeships, security clearances, and industry certifications rather than on conventional interviews and networking alone; and (d) team structures organized around technical expertise and process ownership rather than open-ended collaborative work. Each of these features interacts with autistic ways of engaging, often favourably in terms of task fit, and often unfavourably in terms of access. Advanced manufacturing therefore deserves examination in its own right, not as an instance of “STEM employment” broadly conceived (Raymaker et al., 2023).

For autistic college students, however, the gap between participation and equitable advanced-manufacturing employment remains shaped by longstanding shortcomings in how institutions prepare students for work and connect them to opportunity (Hora, 2020). C. Anderson and Butt (2017) documented early evidence that autistic students entering higher education often feel academically prepared but remain under-supported in the social, executive-functioning, and transition-planning domains critical to employment readiness. Cox et al. (2021) further demonstrated that institutional norms, policies, and accommodation structures create subtle but cumulative barriers, especially around disclosure, accommodation processes, and implicit social expectations. Trusson and Travers (2025) likewise show that careers services, internships, and placement programs remain bureaucratically siloed, weakly sustained across the student lifecycle, and rarely tailored to autistic students’ self-identified strengths or needs, while Vincent (2020) found that institutional supports are often fragmented, inconsistently implemented, and insufficiently aligned with career preparation. However, mentoring is among the most consistently reported enablers of autistic students’ persistence and career development. Yet Morris et al. (2024) scoping review found peer‑mentoring programs “highly variable”, with most offering “an individualized approach rather than a structured curriculum” and only “general support in navigating college” (p. 27). Notably absent is any account of what effective mentorship consists of, emotional support, navigational guidance, or industry knowledge, notwithstanding its role in sector-specific pathways. Notably absent is any account of what effective mentorship consists of, emotional support, navigational guidance, or industry knowledge, particularly within sector-specific pathways. This gap directly informed our protocol: mentorship was included as an explicit topic not to establish its importance, which the literature already suggests, but to examine its composition and function within advanced manufacturing specifically.

Taken together, this literature points to an urgent need for intentional, neuroinclusive pathways into advanced manufacturing specifically, a sector in which autistic strengths appear unusually well matched to task demands, but whose sensory environments, credentialing pipelines, and team structures generate barriers unlike those documented in general employment scholarship. To address this gap, the present study uses focus groups to examine autistic college students’ firsthand perspectives on accessing, preparing for, and navigating roles in advanced manufacturing. Specifically, our research questions ask:

RQ1: What are the current enablers and barriers for autistic college students pursuing a career in advanced manufacturing?

RQ2: What are the ways advanced manufacturing education pathways can be more inclusive and attuned to autistic college student needs?

## Methods

### Participants

Potential participants were invited through outreach facilitated by local disability services, which shared study information with their networks. Interested individuals responded directly. From this pool, the research team first screened participants who met the inclusion criteria, including being currently enrolled in, or having recently graduated from, a U.S. college or university STEM program at the undergraduate or graduate level, being age 18 or older, and being autistic. Following eligibility screening, the research team used purposive selection to prioritize participants whose disciplinary backgrounds or prior experience were particularly relevant to advanced manufacturing and semiconductor career pathways, including from Electrical or Computer Engineering major or having relevant experience of coursework, research, hands-on project, internships, or employmentsuch as an Electrical or Computer Engineering major, or relevant experience in coursework, research, hands-on projects, internships, or employment. When forming the three focus groups, we considered participant availability while seeking diversity across sex, educational stage, disciplinary background, and prior exposure to advanced manufacturing. This approach was intended to capture a range of perspectives and to address the full pathway from exposure to advanced manufacturing careers through preparation, recruitment, and early workplace transition.

Focus groups were chosen over one-to-one interviews because they are well suited to surfacing shared experiences and collective sense-making, allowing participants to build on and contrast one another’s accounts in real time and producing a clearer view of which barriers and enablers are widely shared versus context-specific (Krueger & Casey, 2015; Wilkinson, 1998). To preserve these analytic advantages while remaining accessible to autistic participants, sessions were intentionally small (4–6 participants), conducted via Zoom with flexible chat/audio/video engagement, and facilitated using a structured, concrete protocol that minimized ambiguity and social-communicative demand.

Prior to the focus groups, each selected participant received their study information via an online form outlining the sessions, the voluntary nature of participation, and assurances that identifying information would be kept confidential. Participants were informed that they could withdraw their interview data partially or fully for one week after the interview. Full IRB/ethical review was received from the University of Florida before the study began. The research team adhered to the approved protocol governing recruitment, communication, inclusion criteria, accommodations, and voluntary withdrawal and data removal. Facilitators also attended to participants’ wellbeing by offering breaks, normalizing the option to pause or pass, and signposting external support resources at the close of each session. Participants received a $60 Amazon gift card upon completion. Table 1 shows a breakdown of participant demographics.

**Table 1.** Summary of participant demographics (*N* = 16).

ID	Focus Group	Age Range	Gender	Formal/ Self ID	Major	College Year	Degree Level	Semiconductor Related Exposure
P1	Gp1	30–34	Male	Formal Diagnosis	Biology / Life Science	Graduated	Bachelor’s degree (BA/BS)	Coursework and lab project
P2	Gp1	25–29	Male	Formal Diagnosis	Electrical / Computer Engineering	PhD student	Doctoral degree (in progress)	Coursework, internship, research, and employment
P3	Gp1	35–39	Male	Formal Diagnosis	Environmental Science / Earth Science	Master student	Master’s degree (in progress)	Employment
P4	Gp1	35–39	Male	Formal Diagnosis	Physics	Senior (Undergraduate, 4th year)	Bachelor’s degree (in progress)	Internship
P5	Gp1	25–29	Male	Formal Diagnosis	Chemistry	Senior (Undergraduate, 4th year)	Bachelor’s degree (in progress)	Employment
P6	Gp2	25–29	Male	Formal Diagnosis	Electrical / Computer Engineering	Master’s student	Master’s degree (in progress)	Coursework and internship
P7	Gp2	25–29	Male	Formal Diagnosis	Electrical / Computer Engineering	Senior (Undergraduate, 4th year)	Bachelor’s degree (in progress)	Coursework and research
P8	Gp2	25–29	Male	Formal Diagnosis	Electrical / Computer Engineering	Junior (Undergraduate, 3rd year)	Bachelor’s degree (in progress)	Coursework and internship
P9	Gp2	18–24	Male	Formal Diagnosis	Electrical / Computer Engineering	Senior (Undergraduate, 4th year)	Bachelor’s degree (in progress)	Employment
P10	Gp2	25–29	Male	Formal Diagnosis	Electrical / Computer Engineering	Junior (Undergraduate, 3rd year)	Bachelor’s degree (in progress)	Coursework, research, and internship
P11	Gp2	18–24	Female	Formal Diagnosis	Electrical / Computer Engineering	Senior (Undergraduate, 4th year)	Bachelor’s degree (in progress)	Coursework and internship
P12	Gp3	25–29	Male	Formal Diagnosis	Engineering Technology	Graduated	Bachelor’s degree (BA/BS)	Research and employment
P13	Gp3	30–34	Male	Formal Diagnosis	Mechanical Engineering	Senior (Undergraduate, 4th year)	Bachelor’s degree (in progress)	Internship
P14	Gp3	25–29	Male	Formal Diagnosis	Electrical / Computer Engineering	Graduated	Bachelor’s degree (BA/BS)	Coursework and lab project
P15	Gp3	25–29	Male	Formal Diagnosis	Engineering Technology	Junior (Undergraduate, 3rd year)	Bachelor’s degree (in progress)	Coursework and internship
P16	Gp3	18–24	Female	Formal Diagnosis	Engineering Technology	Sophomore (Undergraduate, 2nd year)	Bachelor’s degree (in progress)	Internship

All participants (*N* = 16, 100.00%) reported having a formal diagnosis. Ages ranged from 18 to 39 years, with 56.25% aged 25–29 (*n* = 9), 18.75% aged 18–24 (*n* = 3), and 12.50% each aged 30–34 and 35–39 (*n* = 2 in each group). The sample identified predominantly as male (*n* = 14, 87.50%), with two female participants (*n* = 2, 12.50%). Participants were drawn entirely from STEM disciplines. Half majored in Electrical or Computer Engineering (*n* = 8, 50.00%), followed by Engineering Technology (*n* = 3, 18.75%). The remaining participants majored in Biology/Life Science, Environmental Science/Earth Science, Physics, Chemistry, and Mechanical Engineering (*n* = 1 each, 6.25%). Most were current undergraduate students (*n* = 10, 62.50%), including six seniors, three juniors, and one sophomore. Three participants (18.75%) had already graduated with bachelor’s degrees, while the remainder were enrolled in graduate programs, including master’s (*n* = 2, 12.50%) and doctoral (*n* = 1, 6.25%) studies. All participants expressed interest in, and had background or experience related to, the semiconductor industry, including courses, internships, job roles, and research involvement.

The protocol was organized into four sequential blocks, each opened by a concrete, low-ambiguity question and followed by structured prompts: (1) background and current program of study (e.g., “Tell us about your degree program and what drew you to it”); (2) experiences of preparing for technical careers (e.g., “What experiences, coursework, labs, internships, have most prepared you for the kind of work you want to do?”); (3) barriers and enablers in pathways toward advanced manufacturing (e.g., “When you think about working in advanced manufacturing, for example, semiconductor fabrication or microelectronics, what do you anticipate would support or get in the way of your success?”); and (4) closing recommendations (e.g., “What is one thing colleges or employers could change to make these pathways more inclusive?”). This sequencing moved from concrete description towards broader reflection, reducing social-communicative demand early in the session and allowing rapport to develop before more open-ended discussion. Facilitators used structured follow-up prompts within each block to encourage discussion of advanced manufacturing education, career aspirations, and encountered barriers or enablers, while participants remained free to shape the direction of conversation within and across blocks. The full question set and prompts are presented in Table 2.

**Table 2.** Semi-structured themes discussed during the focus groups.

STEM Interest and Career Aspiration	What aspects of the semiconductor field interest you the most?
	What is your personal career goal in this field?
	One thing that helps you most when learning about semiconductor fabrication during college (e.g., hands-on lab, lecture, internship, etc.)?
Job Searching Process	What was your experience when searching for internships or entry-level positions? Can you describe what the process was like?
	Have you ever participated in a career training or internship before? What was that like?
	From your perspective, what challenges might autistic individuals encounter when pursuing jobs in the semiconductor industry?
Support	What kinds of support did you have?
	What types of support or resources would help you feel more confident as you transition from schools into the workforce?
	What would make a mentor helpful?
	What should employers or faculty understand better?

### Procedures

We conducted three focus groups with 16 autistic college students, each lasting 90 minutes. Sessions were held on Zoom, and participants were drawn from nine U.S. states. Three members of the research team (the first 3 authors of this paper) facilitated focus groups: one led the discussion and introduced the purpose of the session, while the second provided support and monitored engagement. Three members of the research team (the first three authors) facilitated the focus groups: one led the discussion and introduced the purpose of the session, while the others provided support and monitored engagement. Sessions were recorded using Closed Captions in Zoom, and audio was also recorded to ensure accurate transcription. Facilitators remained attentive to power dynamics in research conducted by faculty and research staff with participants and worked to minimize them by using accessible language, inviting alternative perspectives, normalizing silence and pass-options, and reinforcing participants’ expertise on their own experiences.

### Thematic analysis

Automatic transcriptions were reviewed and checked for accuracy against the audio recordings, producing verbatim transcripts. The data then underwent thematic analysis structured by Braun and Clarke’s (2006) six phases (familiarization, coding, theme generation, review, definition, and reporting), operationalized through a codebook approach: because our research questions were defined a priori, we employed a primarily deductive strategy rather than the reflexive approach Braun and Clarke (2019) describe for fully inductive work. This provided a structured framework for categorizing responses and identifying patterns related to enablers, barriers, and strategies for more inclusive education pathways.

Initially, data from each focus group were analysed separately before being merged into a single dataset. A coding manual, including clear definitions for each code, was developed to ensure consistency and precision. Candidate themes were developed by clustering codes and reviewing the extracts within each cluster for coherence and distinctiveness. The first two authors met to review alignment between codes and data; disagreements over coding or theme placement were resolved through discussion and by returning to the verbatim transcripts, with each resolved decision documented in the coding manual to maintain consistency across coders. In line with a codebook approach, we did not seek statistical inter-rater reliability; consistency was pursued through shared definitions, piloting, and documented adjudication.

The coding manual was drafted by the second author following initial familiarization with all three transcripts, then piloted on one transcript by one team member and refined through discussion before full coding began. Deductive codes derived from the research questions (e.g., barriers, enablers, pathway recommendations) structured the first coding pass and themes that did not fit these a priori categories were retained as inductive codes and reviewed against the research questions during theme refinement, so that unanticipated findings were neither forced into the existing framework nor discarded. During theme review, inductive codes were examined in their own right rather than subsumed under the research-question categories, which allowed unanticipated findings to shape the final thematic structure. In line with Braun and Clarke’s (2019) emphasis on researcher reflexivity, we note the team’s positioning in relation to the data. The analysis was conducted by researchers with professional backgrounds in autism research and clinical practice, educational technologies, immersive technologies, advanced manufacturing fields and also included self-advocate autistic researchers. We treated this positioning as an analytic resource rather than a source of bias to be eliminated, in that team members’ familiarity with autistic students’ experiences informed coding sensitivity, while coding decisions were disciplined by the manual, piloting, and documented adjudication described above.

Once coding was complete, one member of the research team independently reviewed all text segments associated with each code and generated detailed summaries. These summaries were then collaboratively reviewed by another team member to help resolve discrepancies and ensure coherence in interpretation. Following Braun and Clarke (2021), we do not claim data saturation, which rests on assumptions incompatible with thematic analysis, namely, that meaning resides in the data. Instead, we judge the dataset adequate for our aims: three focus groups of 16 participants produced rich, patterned accounts relative to the specificity of our two research questions, and our claims are bounded accordingly. We do not claim that our themes exhaust the experiences of autistic STEM students as a population.

Finally, throughout the study, we adhered to the COREQ (Consolidated Criteria for Reporting Qualitative Research) checklist (Tong et al., 2007) to ensure methodological transparency and completeness. Procedures, data collection, and reporting were reviewed against the 32 COREQ items, supporting clarity, rigour, and reproducibility of the qualitative analysis.

## Results

Based on the research questions, analysis of the focus‑group data yielded six overarching themes with associated sub-themes. For RQ1, themes captured participants’ reported experience along the education-to-industry pathway, including how they accessed knowledge and hands-on learning opportunities, navigated career preparation and self-presentation, encountered barriers during the transition, and relied on often fragmented institutional, informal, and digital supports. Sub-themes further specified learning resources, experiential opportunities, application preparation, workplace norms, sensory and emotional requests/demands, and the use of AI-based tools.

For RQ2, themes focused on participants’ perspectives and expectations regarding inclusive pathway design and mentorship. These included the creation of sensory-friendly and structured learning environments, opportunities to bridge theory and practice through hands-on experiences, practical career preparation workshops, and mentorship models that provide autism-aware guidance, psychological preparation, and industry-specific insights. Together, this thematic structure informed the organization and interpretation of findings. Representative quotes illustrate how each theme is grounded in participants’ lived experiences of pathways into the advanced manufacturing industry.

### RQ1: what are the current enablers and barriers for autistic college students pursuing a career in advanced manufacturing?

#### Theme 1: accessing knowledge and skills through diverse learning pathways

Participants described gaining semiconductor-related knowledge and practical experience through formal and informal pathways. Two subthemes were identified: learning resources and hands-on experiences.

Participants used classes, reading materials, peer study groups, and online platforms such as Google, YouTube, and TikTok. As one participant explained:

Outside of class, I like to practicalize things on my own, so I like to spend time watching, do-it-yourself, videos, tutorial videos on YouTube, TikTok, I like to read into articles, try to connect what I’ve learned lectures to real-world applications.

(P9, Gp2)

Some also sought support from professors, mentors, or professional networks. One participant noted, “I have a couple of people who I’m looking up to. They helped me, motivated me to see the next step that I’m going to take … I could directly ask questions and understand how each tool really worked” (P8, Gp2).

Participants also emphasized applying learning through laboratory projects, troubleshooting, and internships. One participant highlighted the value of combining theory and practice, stating “it’s just a combination of theory and hands-on experience to help me build some strong foundation in semiconductor manufacturing” (P8, Gp2). Another described learning by doing:

It was frustrating to me at first, because the system didn’t really work the way I wanted. But after spending time testing, observing, I was able to bring it up running properly. That taught me patience and paying attention to details. That’s how I somehow managed to approach all programs outside of class. I try to actively explore internships and workshops … the actual hands-on experience that teaches you the most of the things that make someone more effective in a real job.

(P15, Gp3)

#### Theme 2: navigating career preparation and self-presentation

Participants reported three main strategies for obtaining internships and employment: self-directed career navigation, preparation of job application materials, and the ability to articulate and market themselves. Participants often began by searching for positions online and using professional networks. One participant said, “I researched about the company, the labs, specifically work with semiconductors … I reached out to some of my professors and some professionals working at those companies” (P1, Gp1). They then focused on application materials and interviews. One participant described tailoring documents to a position: “I … tailoring my applications, my CVs … I was asked to submit my CV and an executive summary … I filled out the application form, I submitted it, and I attached my CV, so I was called for interview after a week” (P1, Gp1). Another sought peer help with interview preparation: “I had to get a friend that was in the field to do a mini-interview section for me” (P16, Gp3).

Participants stressed the importance of clearly presenting relevant skills and prior experience. One participant linked successful self-presentation to an internship offer: “I focused on presenting my experiences, selling myself very well, so I went in, and the interview was very successful, and that was how I got the internship” (P7, Gp2). Another emphasized precise wording in applications: “one thing that helped me was learning how to present my skills clearly … put a specific word really increased the number of responses I received” (P11, Gp2). Participants also argued that effective CVs and interviews require concrete examples:

You’re supposed to show interest, show them you have knowledge in this … give them examples, let them know you have interest or experience … Don’t just give them a one-line sentence, except if they ask you to. You don’t expect interviewers to tell you what they expect from you. They’re coming to that interview to see what you can give to them.

(P16, Gp3)

#### Theme 3: transition barriers from education to industry

Many described repeated unsuccessful attempts to secure positions, highlighting four barriers: information opacity, lack of prior experience, implicit workplace rules and social norms, and sensory and emotional overload. First, participants described limited information about job postings, resume development, and interview preparation. One participant noted, “It was very challenging at first, because there was not much information” (P7, Gp2). Others described uncertainty about writing a competitive resume, with one participant explaining, “by the time I was applying for my internship … basically I didn’t know how well to write a CV’ (P10, Gp2). Another said, “My CV application got turned down three times and I had to take extra classes which helped build my CV better’ (P4, Gp1). Participants also struggled with unclear interview formats and broad behavioural questions. One participant said:

One major challenge was not knowing what type of interview it would be. Whether it was behavioral, technical, or problem solving. A lot of companies don’t give details, so I ended up preparing for everything and feeling unsure. A lot of difficulty was unrelated to the actual engineering tasks … questions like, tell me about yourself, or very long behavioral questions can be overwhelming … Lack of clear instruction also made it hard … The frustrating parts were unclear expectations, very broad questions, and uncertainty about the whole interview format.

(P11, Gp2)

Another participant described difficulty interpreting what interviewers actually wanted:

I think one of my biggest challenges in interviews when I get questions like, tell me about yourself, and I didn’t know the interviewers were expecting small talks, so I’ll just give you a brief and factual answer … I don’t know, to them, it was seeming like I was showing lack of interest that I didn’t care. That was an issue. This is just me being plain, being myself …

(P16, Gp3)

Second, lack of prior industry experience was a major employment barrier. As one participant noted, “I try getting a job too, but they always talk about prior experience’ (P4, Gp1). Even strong conceptual knowledge could not compensate for the absence of hands-on practice. One participant reflected:

Prior experience is very important, because while I was trying to get a place where I can do my internship, I was asking about career experience working with companies, or things related to semiconductors … I have some videos I have watched on YouTube, and other things I have read about that. I actually didn’t try them practically. So, while in the interview, I was demonstrating a lot of things, it was really a tough moment for me.

(P6, Gp2)

Third, participants faced challenges once in workplace settings, particularly around unwritten rules, communication, and workload management. One participant summarized that “communicating with colleagues, understanding unwritten rules, and navigating team dynamics can be tough” (P13, Gp3). Another described being overwhelmed by workload demands:

I was supposed to be working 7 hours in a day for the shift, and I really had a handful of things. I had study about some things, so the time was just way too much, and I couldn’t manage it properly, so that was one of the things that made it so hectic for me.

(P1, Gp1)

Finally, participants described emotional and sensory hurdles across applications and workplace participation. One participant shared, “rejection for me is always tough, because I’ve been rejected quite a few times actually, but I still kept the mindset, kept going” (P11, Gp2). Another detailed sensory overload in manufacturing environments:

Whether you like it or not you have to use these things, and this involves bright lights, constant machine noise … .you have to wear some goggles to protect your eyes from these slides and this machine noise, but there was a time I had this experience that I don’t know what happened, but it was just too much for me to handle, and I kind of blanked out (P16, Gp3).

#### Theme 4: reliance on fragmented and unstructured support systems

Participants relied mainly on fragmented and unstructured supports when seeking career guidance, institutional and informal supports, supplemented by digital and AI-based tools. A few participants used university career centres for resume development. As one participant said, “I seek advice from my university career council, so it actually helped me focus more on emphasizing, integrating my previous projects and achievements, rather than listing tasks’ (P9, Gp2). More often, however, participants preferred support from family, friends, and professional contacts because these felt more approachable and comfortable:

I can say two people I know who are already professionals, so I had guidance through them, and it was a very impactful journey … I learned more from my friends and the connection, because they are people I was more comfortable with, so I could easily feel free and learn more and ask questions in areas I didn’t know, or I lacked understanding.

(P1, Gp1)

These informal networks provided practical advice, such as building a LinkedIn presence or optimizing resumes for Applicant Tracking Systems (ATS). One participant modelled his CVs on those of professionals in his network: “at first I’d ask people who are professionals, who are better than me, I’ll just borrow them, their CVs and resumes and go through” (P8, Gp2).

Participants also used digital and emerging AI tools to refine resumes. One explained:

I tried to look for online resources that can be able to scan a CV and resume to make sure they are ATS friendly. Some platforms would suggest for you fixes on, or areas where you have weaknesses in your CV and resume.

(P8, Gp2)

Another said, “after the AI was in place already, I had to research using AI, and it really did some teaching on me, and that’s how you know how to write a good CV” (P10, Gp2). Social media spaces such as Reddit were also used for peer critique. One participant described the process of “roasting” a resume to identify problems:

Reddit, several people learn how to build a CV from there, because when you think your CV is complete from your own perspective, you just take it there and just type, roast my resume, or roast my CV, and people just start pointing out the faults or things that not are supposed to be there, because people there are all professionals, and from there, you learn from the mistakes, and you adjust, and you correct things that you’re supposed to correct (P7, Gp2).

### RQ2: what are the ways advanced manufacturing education pathways can be more inclusive and attuned to autistic college student needs?

#### Theme 5: designing holistic, inclusive learning environments that foster engagement, skill development, and readiness for transition

This theme captures participants’ expectations for environments that respond to learning and sensory needs while preparing students for transitions into the advanced manufacturing workforce. Four subthemes were identified: inclusive and sensory-friendly environments, structured social engagement, hands-on learning bridging theory-practice gaps, and practical career preparation workshops. Participants emphasized equitable, inclusive, and sensory-friendly environments where autistic learners can engage meaningfully with peers and learning content. As one participant reflected from their current work experience:

It should be something that wouldn’t be too crowded, and also, there should be a room where everyone’s opinion will be respected, and every opinion will count. There should be no disregard for anyone’s idea … the most important thing for me to succeed is actually having an inclusive and important environment where diverse perspectives are valued, and everyone has equal access to mentorship, resources, and opportunities in the industry.

(P5, Gp1)

Participants also identified cognitive pacing as central to inclusion, noting the need for adequate time to process new information and social situations. One participant described feeling overwhelmed during their recent summer internship and said, “let me get to be comfortable in this environment, just give me a bit of time … to process all of this information, to process meeting a lot of people at once … Let’s prepare mentally, so we don’t have a breakdown” (P16, Gp3). To support engagement, participants suggested structured, low-pressure social activities rather than unstructured networking. Group-based activities such as board games and brainstorming tasks were recommended to “keep a lot of persons together, make them happy and keep them engaged” (P6, Gp2). Another participant explained that such group-based gaming is a “very important opportunity for autistic students to practice social interaction, teamwork and communication” (P1, Gp1).

Participants also prioritized hands-on laboratory experiences that translate theoretical knowledge into industry skills. Practical opportunities in fabrication and troubleshooting were especially valued, as one participant reflected on his hands-on experience on a lab project:

There should be hands-on experiments or lab activities, where participants can observe materials, fabricate simple devices … improving on my fabrication techniques. Also, for people who wants to work in the industry, learning how to troubleshoot or solve, real-world application problem is very important … The most important thing to be succeed in the industry is to develop strong practical skills, enhance some experience with material devices.

(P1, Gp1)

Participants further emphasized career preparation workshops focused on job searching, strengths and interests mapping, CV building, and mock interviews. One participant reflected on her own experience and highlighted the need for explicit guidance on navigating job-search platforms and avoiding scams:

The workshop should include opportunities where they can apply for jobs … this is how you should go about using LinkedIn. If you want to use websites, or you want to apply to companies directly, this is how you should do it … if you just give them a theory aspect, by the time they get back into the world, they might still be confused, because there’s a lot of websites there, and there’s a lot of job scams as well. I’ve experienced quite a few ….

(P16, Gp3)

Others emphasized helping students identify and articulate their strengths in ways that align with industry expectations. One participant said, “knowing their skills, if he or she should make a list of hands-on skills, projects, and accomplishments. When students understand what they offer, it’s easier to speak about themselves confidently” (P11, Gp2). Another participant wished to know more “tips on how to write a captivating resume that the industry would like to have you, and also how to market yourself” (P2, Gp1). Mock interviews and role-play were also seen as valuable confidence-building tools. Drawing on her own internship interview preparation, P11 (Gp2) suggested starting from having mock interviews with friends:

Mock interviews or role-playing networking situations helps students get comfortable with the questions and the setting before the whole routine … maybe starting small practice, choosing yourself to peers or mentors in low-pressure settings, gradually increase exposure to presentations or interviews.

#### Theme 6: mentorship as a critical bridge between learning, neurodiversity-aware support, and career progression

Beyond environmental conditions and learning design, mentorship was recognized as a key mechanism for navigating transitions into advanced manufacturing careers. Mentors were expected to support psychological preparation, provide autism-aware guidance, and offer industry insights that facilitate career growth. Participants emphasized that successful transitions require technical knowledge and mental and emotional readiness for workplace challenges. One participant said, “They need to be mentally prepared … They gotta know that they’re gonna face a lot of challenges, and ought to know the kind of things to expect” (P12, Gp3). Mentorship was valued as a way to help autistic individuals “build confidence step by step” through “preparation, self-awareness, small successes” and a “positive mindset” when entering a new field (P11, Gp2). With guided preparation, students are “not shocked” but “feel comfortable enough to work and also be effective in what they do” when challenges arise in the workplace (P12, Gp3).

Participants also expected that mentors must be prepared to provide autism-aware support and avoid reinforcing stigma. As one participant explained:

There’s a kind of safety that mentor is prepared. They have to be able to prioritize the students that are autistic so that the work environment suits them, and so to enhance their productivity and their deliveries. Proper interviewing of the people that’s going to be the mentors, so they won’t have to face stigmatization (P12, Gp3).

Participants also wished to receive concrete, visual, step-by-step guidance. One participant explained:

Having someone in the field who could break things down in a very concrete and visual way made a huge difference for me. Having that clear instructions, consistent routines, and being able to see examples of good practices really supported my learning style.

(P11, Gp2)

Using visual diagrams, flowcharts, and “break complex topics or tasks into little manageable pieces” was seen as helping autistic learners manage complexity more effectively (P16, Gp3). Finally, mentors were expected to offer industry knowledge and career guidance often absent from formal education, including clearer insight into industry structures, emerging trends, and career pathways. One participant explained: It would be really valuable to introduce a short segment about industry strengths and structure. For example, which companies focus on packaging, which focus on chip design, or who handles fabrication versus testing. So, highlighting current trends, like advanced packaging, wafer-level testing, or high-performance computing applications will give students that context and motivation. And it also helps them see potential career paths and where their skills would fit in (P11, Gp2).

Two participants also wanted help learning how to “position themselves” (P2, Gp1) and “stay adaptable in a rapidly evolving industry” (P3, Gp1). For students without prior internship experience, mentors were seen as especially valuable in recommending high-quality programs for gaining practical exposure. As one participant reflected:

I think it would be important to ask if there are internship or programs that they recommend for gaining a real-world experience. It would be very important, because the starting point is very important to career growth.

(P1, Gp1)

Taken together, the six themes describe not six separate experiences but a single interacting pattern. The barriers participants encountered (Theme 3) were rarely the product of any one stage. For example, the information opacity in career preparation (Theme 2) compounded the sensory and social demands of the transition itself, and the absence of structured guidance at both points drove the reliance on fragmented informal and digital supports documented in Theme 4, supports participants turned to precisely because formal systems had not met them. In this sense, Theme 4 is best understood as a consequence of Themes 1–3 rather than a parallel finding. The two RQ2 themes function as the corresponding response, that is inclusive, sensory-aware, hands-on learning environments (Theme 5) address the conditions under which knowledge is acquired, while mentorship (Theme 6) addresses the interpretive gap (implicit norms, self-presentation, psychological preparation), that no curriculum change alone resolves. Overall, therefore, the research questions are answered jointly. RQ1 identifies where the pathway fails (preparation, transition, and the informal patchwork those failures produce), and RQ2 identifies the two intervention points, environment design and mentorship, that our participants’ accounts indicate would do most to fix it.

## Discussion

This study offers the first in-depth exploratory examination of autistic college students’ experiences navigating education-to-career pathways into advanced manufacturing, a sector whose persistent talent shortages, and substantial public investment, have positioned it as a strategic priority for workforce development, yet one that has remained almost entirely absent from the autism and employment literature to date. Our findings tend to emphasize the critical importance of centring autistic voices. Our findings underscore the importance of centring autistic voices in discussions of disability inclusion, echoing broader critiques of higher education and workplace support systems (Holmes, 2024; Nerren, 2022). Our research questions examined the current enablers and barriers for autistic college students pursuing careers in advanced manufacturing. Our participants described accessing knowledge and skills through a range of formal and informal pathways, including university courses, peer networks, online platforms, and hands-on laboratory experiences. This pattern was directly visible in our data: participants in all three groups described information opacity and implicit workplace rules as their most consequential obstacles (Theme 3). These findings highlight the proactive strategies autistic college students employ to develop technical expertise, aligning with prior evidence that autistic learners are highly motivated to engage with domain-specific content when given accessible and self-directed learning opportunities (Leach & Duffy, 2009). At the same time, students emphasized persistent challenges in transitioning from education to industry. Systemic barriers included opaque application processes, unclear expectations in interviews, limited prior work experience, and the emotional and sensory demands of industrial workplaces. These experiences reflect many structural limitations, particularly the fragmented and siloed nature of institutional support, which leaves students navigating complex transitions with inconsistent guidance (Cox et al., 2021; Longville-Carr, 2024).

Our participants’ reliance on fragmented institutional, informal, and digital supports (Theme 4), including their turn to AI-based tools, mirrors Bjartmarsdóttir and Newbutt’s (2026) autoethnographic account of neurodivergent higher education experiences, in which limited access to formal accommodations shifted the burden of support onto the individual. Both accounts converge on the same finding, that is, when institutional support is fragmented, autistic students assemble their own patchwork of informal and digital workarounds, reinforcing the case for structured, integrated supports rather than ad hoc accommodation. Moreover, participants expressed a reliance on informal and digital supports, including family, peers, professional networks, and AI-driven tools for resume building, underscores the inadequacy of existing institutional structures. While students creatively leveraged these resources to compensate for gaps in university-provided support, their experiences reveal how reliance on self-directed strategies may inadvertently exacerbate inequities, privileging those with greater digital literacy, social confidence, or external support networks. This observation resonates with Trusson and Travers (2025) finding that university employability support often diminishes off-campus responsibilities, leaving autistic students underprepared for real-world workplace expectations. This observation resonates with Trusson and Travers (2025) finding that university employability support often declines during off-campus placements, leaving autistic students underprepared for real-world workplace expectations. Our data extend this finding by demonstrating how fragmented supports not only impede skill application but also generate emotional strain, highlighting the interplay between structural barriers and psychosocial outcomes (Koops et al., 2025; Toor et al., 2016). Specifically, found that support programs for autistic university students positively impact well-being, academic achievement, and retention. Mentoring interventions were particularly effective when they focused on individuals’ strengths, interests, and challenges. Similar to our findings, Toor and colleagues suggest that future programs are tailored to the specific needs of autistic undergraduates and developed using co-design principles, emphasizing supports that bridge the gap between college education and work preparedness.

Our second research question explored how advanced manufacturing education pathways can be more inclusive and responsive to autistic college student needs. Participants discussed the importance of holistic, sensory-friendly learning environments, structured social engagement, and hands-on, transition-oriented experiences. These findings align with the principle of inclusion and moving beyond isolated disability services towards institution-wide approaches that accommodate neurodiverse learning styles and support functional life‑skills development. This aligns with the findings of Janse van Rensburg and Liang (2025) who suggest a shift in autistic supports, that extend beyond disability services to include peers, instructors, support staff, faculty, and the institution as a whole. These findings align with calls to move beyond isolated disability services towards institution-wide approaches (Janse van Rensburg & Liang, 2025) encompassing peers, instructors, support staff, faculty, and the institution as a whole. Here the authors argue for restructuring institutions to promote autistic culture, provide autonomous support and accommodations, and foster environments where students can thrive academically and socially. Our participants also highlighted the need for structured, low-pressured social activities and clear cognitive pacing; all of which highlight how traditional educational settings often fail to anticipate and mitigate environmental and social stressors for learners.

Theme 6, Mentorship emerged in our data not as a generic good but as a specific bridge across learning, support, and career progression (Theme 6), with participants distinguishing psychological preparation, autism-aware guidance, and industry insight. Mentorship also emerged as a particularly important aspect for supporting our autistic college students’ career progression. Our findings suggest the value of mentors who provide autism-aware guidance, practical industry insights, and psychological preparation for workplace challenges. Importantly, mentorship was not framed as a remedial intervention but as a bridge between students’ existing strengths and industry requirements (Szechy et al., 2024). That mentorship matters for autistic students is consistent with prior literature; our contribution is to specify its anatomy. Participants did not describe mentorship as a single supportive relationship but as three distinct functions, psychological preparation, autism-aware guidance, and industry-specific insight (Theme 6), and attributed transition failures to the absence of one or more of these functions, not of mentorship per se. This differentiation offers the structure that the “highly variable” programs in Morris et al. (2024) review currently lack.

The double empathy problem holds that social difficulties involving autistic individuals arise not from deficits within the autistic person but from mutual misunderstandings between autistic and non-autistic people (Milton, 2012). Our findings instantiate this at the education-to-industry boundary that participants’ accounts of decoding unwritten social expectations (Theme 3) show the DEP operating precisely where workplace norms remain implicit; a link prior literature has theorized but, to our knowledge, not traced through autistic students’ own accounts of advanced manufacturing pathways. Our findings instantiate this at the education-to-industry boundary: participants’ accounts of decoding unwritten social expectations (Theme 3) show the DEP operating precisely where workplace norms remain implicit – a link prior literature has theorized but, to our knowledge, not traced through autistic students’ own accounts of advanced manufacturing pathways. This reframing places responsibility for repair on both parties and employers must develop more explicit and flexible interaction and communication practices, thereby reducing the psychological pressures reported by our participants and experienced by autistic people more broadly (Szechy et al., 2024).

## Limitations and future work

Although this study offers valuable insight into the enablers and barriers autistic college students face when transitioning from education into the advanced manufacturing workforce, several limitations should be acknowledged. First, most participants were men, which may limit the diversity of perspectives, particularly those of autistic women, who often mask, creating additional challenges in higher education and the workforce (Hull et al., 2020). Second, participants were recruited based on interest and willingness to participate, which may have produced a self-selecting sample and excluded those less willing to share their experiences. Unequal participation within the focus groups is also a limitation. Specifically, Participant 14 from Group 3 provided limited verbal responses, so this individual’s perspectives were underrepresented in the analysis. This limited the range of viewpoints represented in the findings and subsequent thematic interpretation. Although three focus groups were conducted, we did not carry out follow-up individual interviews, which could have provided deeper insight and clarified intended meanings. Though the data were independently coded and analysed by two researchers, thematic analysis is interpretive, and findings may reflect researchers’ subjective perspectives. Future research could build on these findings by using complementary methods, such as individual interviews and large-scale questionnaires, to capture both in-depth and broader, more generalizable data on autistic individuals’ transition into the STEM workforce.

## Conclusion and implications

Taken together, these findings emphasize that inclusive practices in advanced manufacturing education require coordinated, systemic approaches that bridge the gap between academic preparation and workforce readiness. Autistic college students possess rich domain-specific skills and strong self-directed learning strategies, yet their potential is constrained by fragmented institutional support, inconsistent curriculum that does not always meet the visual needs of autistic learners, and poorly structured transitions yet their potential is constrained by fragmented institutional support, curricula that do not always meet the learning needs of autistic students, and poorly structured transitions. More broadly, these findings suggest that progress on autistic inclusion in STEM employment will come not from general calls for neurodiversity in STEM, but from field-specific examination of particular sectors, such as advanced manufacturing, where the match between autistic strengths and task demands can be identified, and where pathways can be deliberately built around the specific barriers each field presents.

## Data Availability

Due to ethical constraints, supporting data are not available.
